# The Refugee Experience of Asylum Seekers in Italy: A Qualitative Study on the Intertwining of Protective and Risk Factors

**DOI:** 10.1007/s10903-021-01296-3

**Published:** 2021-10-20

**Authors:** Amalia De Leo, Paolo Cotrufo, Caterina Gozzoli

**Affiliations:** 1 Department of Psychology, Università degli Studi della Campania Luigi Vanvitelli, Viale Ellittico 31, 81100 Caserta, Italy; 2 Department of Psychology, Università Cattolica del Sacro Cuore, Largo Gemelli 1, 20123 Milan, Italy

**Keywords:** Asylum seekers, Refugee experience, Qualitative research, Risk and resource factors, Psychological experience

## Abstract

This study aims to investigate the criticisms and support factors of the migratory experience of a group of asylum seekers (ASs) hosted in an Italian reception center. Starting from a psychosocial approach that gives importance to the intertwining of the personal history and context, the present study aims to explore the meaning that ASs give to their origins, the relationship of ASs with the host context and with professionals of the refugee centres, along with the representation of the Future. We conducted 27 semi-structured deep interviews with 9 male ASs with an average age of 24,4 years. In line with the research goals, we carried out an analysis of pencil-and-paper content using the interpretative-phenomenological-analysis. Three different types of refugee experience emerged: persecutory, ambivalent and integrated. The three conditions identified can help professionals to better understand the different experiences of ASs, allowing them to develop more effective interventions.

## Introduction

Within the vast debate on migration, the phenomenon of asylum seekers and political refugees is inserted with increasing intensity. Although more than 85% of international refugees are actually hosted in developing countries [1], since the beginning of 2015, more than one million people have crossed the Mediterranean Sea and risking their lives to reach Europe. It is recorded that the number of people who died on this dangerous tract is 7269. Those arriving in Europe are often traumatized by the violence they have suffered, both physically and mentally, during the trip [2].

Italy, due to its geographical position in the Mediterranean Sea, is one of the main landing countries of migrants coming from Libya and Turkey. The estimated number of asylum seekers in 2019 was 43,783—the 81% of the asylum requests have been denied—(Ministry of Interior) but the number of people that live illegally in our territory is still unclear; it is estimated that these numbers are increasing mainly as a result of the application of the Security Decree n. 113/2018, issued in October 2018, which led to a drastic reduction in the attribution of the international protections. In particular, the 2018 Security Decree modified some asylum claim procedures abolishing the humanitarian protection^1^ status, which was frequently granted before the 2018 and transformed the System of protection for asylum seekers and refugees (SPRAR) to the System of protection of refugees and unaccompanied minors (SIPROIMI), excluding asylum seekers and beneficiaries of humanitarian protection status from the second-line reception centers. This led to the irregularization of migrants who were eligible for the humanitarian protection status, aggravating their access to the reception system and leaving them in destitution. The Law also repealed the rules governing civil registration, prohibiting asylum-seekers to register as residence, excluding them from basic health care and social coverage.

These factors transformed the present-day forced migration phenomenon into a priority that needs to be addressed.

Asylum seekers and refugees differ from other migrants, as they have not chosen to migrate but rather have been forced to flee their country, family, community, job and culture [3]. They have not been able to plan their move practically, psychologically and systematically over time. The migratory phase that follows the escape is often unpredictable, tiring, sometimes characterized by an “encounter” with further traumatic experiences [4]. De Micco [5] has argued that the refugee experience is characterized by a suspended and uncertain condition in which the person is hanging between two different societies and forms of culture. Those who cross the “borders” do not cross only geographical borders but also cultural and inevitably psychic ones. ASs, in fact, even when they have reached a new territory, are for a long time anchored at a psychological level in an indefinite space before being able to build a new life. However, this condition of suspension does not necessarily preclude the possibility of feeling part of a wider community, although this is not easy at all.

The outcome of the refugee experience, as with any complex challenge, depends on multiple factors: the meanings ASs give to their origins (homeland, country, family), what they face in the host country, what they expect from the future and the ability and possibility to live in new places in light of the real opportunity of the host context [6].

In the past few years, despite an increasing interest in knowledge about and a definition of the phenomenon of forced migration, there is still a lack of information concerning how asylum seekers understand, organize and describe—in other words represent—their refugee experience. In particular, there are few studies that pay attention to the risks and resources of this experience from a psychosocial point of view and take into account the Italian context even though the country is one of the main players involved in the crisis management, at European level.

In the scientific literature, much attention has been paid for an extended time to the impact of the traumatic aspects of the refugee experience; scholars, in fact, have tried to identify factors that increase the risk of the development of psychological problems in refugees. The most important risk factors will be herein presented.

There is a large body of evidence that indicates that the exposure of refugees to war-related traumatic events has a very high impact on the presence and persistence of mental health problems [7]. Forced migrants share a traumatic past that threatens their integrity and psychic continuity and includes exposure to war-related violence, sexual assault, torture, incarceration, genocide and other forms of threats and personal annihilation [8]. Trauma represents one of the most significant risk factors for depression [9], PTSD [10] and anxiety [11] in refugee populations.

Other studies have led to increased interest in how post-migratory living conditions—daily material and social stressors—influence refugees’ psychological well-being. In particular, the study of Schick et al. [12] highlighted that during this vulnerable time, ASs must also contend with their personal histories, uncertain residence status, isolation from family and social ties, integration into a new society and language barriers. Factors such as the absence of a migratory project, unrealistic or idealized expectations toward the host country and isolation may create conditions of mismatch to the social context and undermine the inclusion process.

According to critical scientific literature, another risk factor can arise from the reception structures themselves (local dispositifs of government of migration), which can produce social suffering and the reiteration of the trauma despite having the exact opposite purpose [13].

In the most recent literature, research which focuses exclusively on trauma or pathology, ignoring the abilities that migrants bring with them on their journeys, has been frequently criticized [14, 15]. In the wake of these studies and distancing ourselves from a reductionist vision that tends to lead the whole migratory experience back to trauma, we will try to adopt a more complex approach that views the refugee experience as a challenge that takes into account both the risks and resources.

Studies that have focused on the resource factors are mostly related to the construct of resilience. Although there is no universal definition of resilience, most scholars use this term to refer to an ability to overcome adverse life events [16]. Other studies have shown the importance of resilience as a protective factor for refugees in resettlement. In this case, resilience is defined as “a dynamic process encompassing positive adaptation within the context of significant adversity” [17]. There is a general agreement according to which higher levels of resilience have been associated with fewer depressive symptoms and other emotional problems [18]. Resilience, in fact, can help asylum seekers and refugees to adjust to their predicament and protect them against the negative effects of trauma [19].

Resilience, however, mainly takes into account individual resources and does not explore in depth the relational dimension that, according to the psychosocial prospective, represents a potentially important protective factor. Bonding can be a valuable type of resource, but if the encounter with the other fails, in particular, the intercultural encounter could turn into an additional source of risk.

In light of these considerations, we will explore the risks and also the resources of the refugee experience by adopting a psychosocial approach that focuses on the intertwining of personal history and context, with particular attention paid to the quality of the bonds [20–23]. In accordance this theoretical perspective and previous studies [3, 6, 24, 25], we will investigate:

- The origins (past);
- The host context and the relationships with the welcoming professionals (present);
- The representation of the times to come (future).

It is important to investigate the premigratory experience and the theme of origins to understand if some positive memory parts survive to the pain that asylum experience lead. As highlighted above, the first post-migratory experience can exert an enormous influence on the mental health of refugees, and the operators play a critical role in providing support and in activating ASs’ resources. Lastly, the way they look forward to the future can help to better understand if there is the possibility of creating new relationships in the host context that may foster the inclusion process.

These three superordinate themes will be further addressed using a bottom-up logic that allows us to detect the emerging themes directly from the words of the participants. In the scientific literature, concerns were raised that the quantitative approach might not accurately capture the realities of migrants, either due to differing sociocultural conceptions, translation problems, or a bias against speaking about themselves and mental health issues [26]. For this reason, to achieve our aims, we decided to adopt the interpretative phenomenological analysis (IPA) [27, 28]. This qualitative methodology gives importance to the personal meanings that participants attribute to their experiences, as well as to the individuals’ inner personal world, and it allows us to explore the deepest aspects of the relational world.

## Materials and Methods

### Aims and Scopes

The study presented here was conducted in collaboration with a reception center (CAS) for refugees and ASs in the city of Brescia, in the north of Italy. Our research aims to explore, through the voices of the ASs, the risks and resources of the refugee experience from a psychosocial point of view. The initial assumption is that in the particular experience of the refugee, ASs experience a condition of extreme fragility that can lead to a condition of psychophysical malaise or, in the most serious cases, can result in mental illness. But despite this, they succeed in activating resources which may also depend on the meanings they give to their origins, on the soundness/stability of the relationships they have established with the Other (in our case, the operators of the refugee center) and on the possibility to think about and build new bonds in the future.

### Methodology

We decided to adopt the Interpretative Phenomenological Analysis (IPA) for our study [27, 28]. IPA is an ideographic research method aimed at exploring human experience in its deep essence going beyond the explicit and the manifest and trying to grasp its latent symbolic meanings. The focus of the analysis is on the exploration of the processes of understanding and meaning of individuals about their experiences and surrounding reality, considering these processes not as a body of objective and static knowledge but as a continuous becoming in the dialogical process of encounter between the individual and his context (physical and social). In the IPA approach the researcher must pay particular attention to the ideological processes of exchange and interaction that take place in the research setting, as they are the basis in the processes of co-construction (and negotiation) of meanings. It follows a double hermeneutic: participants try to give sense to their experiences while the researcher tries to understand the sense given by participants to their experiences. IPA considers the data production phase to be a crucial moment in the knowledge-building process, resulting in close interdependence between data collection and analysis. For this reason, IPA methodology guides the researchers from the implementation of the research design to the data analysis phase.

### Participants

We interviewed 9 African male asylum seekers aged from 18 to 31 (M = 24,4). Three of them come from Nigeria, 2 from Senegal, 1 from Cameroon, 1 from Gambia and 2 from Sierra Leone. A limited number of interviews allows us to study more in depth the experience of the ASs; in fact, a relatively small sample size is the norm in IPA [29]. The strength of the method lies in its ability to provide in-depth idiographic insights that are unique to each individual participant while simultaneously revealing shared themes common across the range of lived experiences among a group of participants [28].

We set the following inclusion criteria to select our participants:

-Males;
-Between 18 and 31 years old;
-People hosted of the same refugee center.

We decided to involve only males because the literature states that there is a substantial difference in the way men and women experience the refuge experience. Regarding the second inclusion criteria, we decided to choose the age’s range 18–31 for the participants because it reflected the average age of the asylum seekers hosted in Italy. Considering the idiographic nature of the IPA, a homogeneous group is usually required, based on certain characteristics that reflect the specificities of the research questions, for this reason we decide to involve only the guests of the same refugee center (Table 1).

**Table 1:** Characteristics of participants

Subject	Sex	Age	Country of origin	Time of arrival in Italy	Number of countries they lived	Civil state	Reason for seeking asylum	Social-juridical Status
1	M	24	Nigeria	4 months	0	Not married	Falsely accused of killing a person	Asylum seeker
2	M	20	Senegal	24 months	1	Not married	Uncle wanted to kill him after he killed his father	Asylum seeker
3	M	18	Sierra Leone	12 months	0	Not married	Escaped from a sect where he was a prisoner	Asylum seeker
4	M	23	Senegal	20 months	2	Not married	Trouble with his family	Asylum seeker
5	M	31	Nigeria	16 months	0	Not married	Persecuted because political activist of the Massob group	Humanitarian protection
6	M	29	Nigeria	17 months	1	Not married	He was discriminated because he was involved in sexual intercourse with a man	Asylum seeker
7	M	29	Cameroon	8 months	2	Not married	For reasons of war in particular for the conflict between the English-speaking and French-speaking parties	Asylum seeker
8	M	20	Sierra Leone	20 months	0	No married	Persecuted by his uncle with whom he had conflicts	Asylum seeker
9	M	26	Gambia	12 months	1	Not married	He was persecuted because his grandmother was a political activist	International protection

### Measures

Semi-structured interviews [30] were used to explore the representations of the ASs with respect to their refugee experience. The excerpts of narrations—which will be presented below—come from a series of in-depth interviews carried out with a group of young people seeking international protection, guests of a reception center located in the Municipality of Brescia, who took part in our study. In particular, we held three sessions with each participant during which we focused particular attention to the understanding of the refugee’s risks and resources in regards migration experience. Specifically, we spent about 7 h with each informant, a total of 63 h of in-depth interviewing in 27 sessions. We can define these encounters as a series of “immersions”, in a certain sense, in the daily life of the subjects involved, that allowed us to investigate in detail the three areas explored. During the first encounter we tried to establish a confidential atmosphere also to test the presence of the cultural mediator who supported us. Once we had ascertained that had been structured a climate of trust, we conducted an in-depth interview with the aim to explore the theme of the origins. In the second meeting, we deepened the relationship with the host context and the operators; while during the third, we investigated the expectations towards the future and their project of life in the new context.

The main questions used to guide the interview were:

1. Would you like to tell me something about your life in your homeland…. What were the conditions in your homeland? What did you do in your country? Can you tell me something about your family?
2. Would you like to tell me something about your journey… Why did you decide to leave? Can you tell me something about the journey?
3. Would you like to tell me something about your arrival… How is your life in Italy? What kind of relationship do you have with operators of the refugee center? What do you expect from the future?

### Research and Procedure

During the asylum request process, ASs are required to deal with people involved in the evaluation of their application such as social workers, judges, and operators. This could push ASs to give an image of themselves and of their migratory experience in order to obtain a residence permit, resulting therefore not very reliable. For this reason, also the researchers could be intended as authoritative figures by AS. To skip this bias, before to start our data collection, we organized a meeting with ASs to describe the aim of the study and the role of the researcher, highlighting that the research would have no effect on procedures for granting asylum. During the first meeting participants were asked to fill out and sign a consent form and an anonymous socio-demographic schedule. All interviews were conducted with the constant supervision of a cultural mediator who was able to conduct the interview in French or in the native language of the guests if necessary. Since most the participants were able to speak Italian or English (as well as the researcher), the cultural consultant was asked to intervene only in cases of misunderstandings. The employees of the reception centre were not present during the interview sessions. The interviews were recorded with the consent of the participants and were fully transcribed from the direct voices of ASs; we reported, instead, the translation of the cultural mediator in the cases where he intervened to support the ASs. The transcripts were read in detail and the individual responses to each discussion area were collected and compared by two researchers [31]. The study was conducted according to the guidelines of the Declaration of Helsinki and approved by the Ethics Committee of Università degli Studi della Campania Luigi Vanvitelli.

### Data Analysis

Data analysis was carried out in parallel with the data collection incorporating the material that gradually emerged from the interviews. In line with the research objectives, the data were analysed using the procedure described in the IPA process [27, 28, 32, 33] aimed at understanding experiences and exploring the process of construction of meaning that individuals use to understand reality, through a subjective perspective, and always considering the sociocultural context in the data interpretation process.

The data analysis has been performed according to a hierarchical categorization system that combines a top-down logic with a bottom-up one. Specifically, through the top-down logic we analyzed the text, defining three superordinate themes: “Origins”, “Relationships with host country and professionals” and “Future”. Subsequently, in order to identify within these superordinate themes, the emerging subordinate, we analyzed the parts of the text considered with a bottom-up logic, as is not yet so well established which specific indicators should be considered on each of the three levels of analysis. Finally, we combined the two kinds of thematic analysis and started an interpretative process to explain the relationship between superordinate and subordinate themes, with the aim of tracing different conditions of the refugee experience.

Data analysis was conducted by two independent reviewers (the agreement was established for each of the pairs of secondary judges A.D. & C.G– C.G. & A.D., and after that, we calculated the mean value). Intercoder reliability was good (Cohen’s Κ = 81%) and was calculated using ComKappa software [34, 35]. Cases of disagreement were considered and discussed until consensus was reached. The main thematic groups emerging from the analysis of the nine interviews will be reported in the results.

## Results

**Fig. 1:**
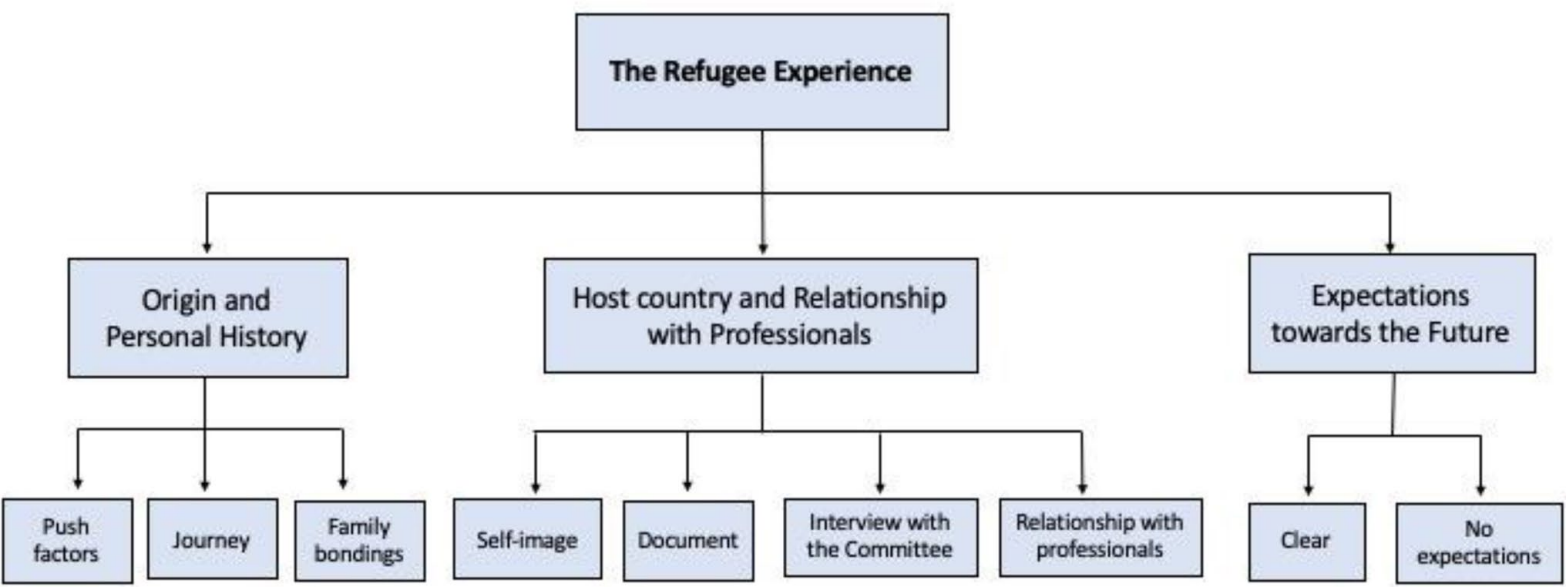
Superordinate and subordinate themes

In line with the general aims and thematic areas explored, the results of the interviews of the nine asylum seekers will be reported. Following that, the recurring and cross-cutting elements with regard to the three different levels considered—origins, landing in Italy and the future—will be presented, looking at resources versus risks (Fig. 1). Considering the IPA guidelines, it will be attempted through a circular logic to connect the most contextual elements emerging from the interviews to the most internal psychological processes, paying particular attention to the meanings that people attribute to their own experience and to relational aspects.

### Origins

The first aspect focuses on the scenarios related to origins (metaphorical places before physical) in which the decision regarding the departure is made; the intent is to better understand what the migrant faced from the first moments of his experience, looking at the risk and resource factors. From the interviews, three subordinate themes emerged: push factors, the experience of the journey and relationship of the ASs with their homeland and family of origin.

The experiences of the participants interviewed were characterized by numerous difficulties and often painful experiences, such as imprisonment, discrimination, violence, wars and persecution. In many cases, such experiences were the primary motivation (push factors) for asylum seekers to escape from their country of origin, thus representing an important risk factor.

In Nigeria the ethnic group is called Biafra; there is so much corruption, so many problems, I was not happy. I was always going to the demonstration, but there were so many problems, someone went to prison, some died, (…)the government intervened in a brutal way, (…)it sent military forces to mistreat people (…) there was violence and so many people died being part of the Massob group." (Nigeria, 31)

The problem was war. (Cameroon, 29)

They catch you! (…) They catch young boys like us and force them to join their sect. (Sierra Leone, 18)

Participants remember their lands of origin as dangerous places which have failed to ensure primary living conditions. Events such as persecution, discrimination, violence and exposure to the brutality of war are the main reasons why ASs decide to flee. These events are certainly destabilizing from the psychophysical point of view and can highly endanger the health of migrants.

The journey towards Italy had an average duration of one year and two months, and it was possible to identify a common denominator to all nine routes narrated: the problematic crossing through Libya. Despite the similarity of the experiences lived, the stories of the participants were very different from one another.

The following two subjects, for example, reported very superficial and fragmented narrations:

During the journey he found himself in a desert and in this desert, there were fighters, desert rebels. They used a gun, a big gun. When you arrive in Libya there is always a big mess, it is not good…In Libya children have guns, ten-year-old children have guns. (Nigeria, 31)

I started the journey as a blind man. I did not have a destination. I did not know where I was going.(Cameroon, 29)

.. I boarded but I didn’t know where to go because I had never seen the sea before. (Nigeria, 24)

Another AS reconstructed in a long story the details of his escape, bringing back a memory that was still vivid and full of painful images:

I ran away because they had caught us. One night, at midnight, they came to pick us up, me and ten other guys. They tied us up and beat us. Since you got caught, you’re considered a ghost, not a person anymore. They danced; it was like a ritual. They took us to the place where they do these ceremonies; it was out of town. Once you leave, you can’t come back. In the morning I was still drinking something I didn’t know. They poured it into some kind of skull, and we fell asleep there, and in the morning when we woke up, we found human flesh. They were also doing this ritual. It was something structured at various times; it would have lasted until the next day. In the night they took one of my companions and told him he would be the first to die. The kid said he knew he was going to die, and we never heard from him again. The house of the ritual was not built in a traditional way, but with palm leaves. We managed to escape because there was a room where they probably killed people, and another room where we had to wait. We were guarded and tied up, but this old man who was watching us was very drunk and not very alert, so one of my companions managed to get away. He showed us how to do it, and we ran into the forest all day long; then we went to another city the next night. (Sierra Leone, 18)

This story is characterized by extreme rationality and accuracy in the details. This description seems to deal with the reasons why the participant was forced to flee; the extreme rationality of the story does not correspond to an emotional dimension, which seems to remain outside the narration. In his words, there is no mention of feelings of fear, anguish or anger. It seems that there was a disconnection between the emotional and cognitive functions of the mind, and that reported story does not correspond to something really lived.

Within our sample, there was also a participant who did not want to share the reasons for his escape. It is the case of a Senegalese man who revealed to us:

I can’t talk about the problem that pushed me to leave Senegal (…)I can’t say, I can’t say what happened. (Senegal, 23)

The difference between the quality of the narrations offers us a greater or lesser elaboration of the experience, and therefore of the activation, or not, of the resources available. The reasons for the departure and the journey are lived out, in fact, mainly as traumatic experiences because of the challenges that ASs have had to face, but among our sample, greater resistance was shown, and these first challenges were managed well.

Another aspect of the refugee experience that emerged from the biography of the ASs is that of the family bonds in the home country. The element that crosses almost every narration of our sample is the discontinuity of the family stories. What the participants reported were fragmented life stories, full of mourning, loss, disease and violence. The act of escape that saves forced migrants is accompanied by a constellation of multiple losses. The survivors of torture often have no choice but to flee suddenly, alone, unable to warn family or friends and without a precise migratory path.

He only has one sister, an older sister. He also has a mother, but she has been ill for a long time, for more than ten years. His father is no longer there, when he got sick you knew he would fail in four years. Right now, the mother is very ill… (Nigeria, 29)

I don’t have a father… I was adopted, the only mother I knew is the woman who adopted me. I don’t know my father, he died in war. And my foster mother died too, I lived with my uncle (Sierra Leone, 20)

When we asked one of the ASs whether he intended to bring to Italy his child born from the union with his partner in his homeland, he replied:

Yes, but I can’t bring them to Italy now because there’s a problem; when I find a contract, I can. (Cameroon, 29)

Inevitably, many of the experiences illustrated, characterized by abuse, forced detachments from the family, an absence of paternal and/or maternal reference figures and bereavement experiences have contributed to the structuring of often fragile and vulnerable identity profiles and to a generation with attitudes expressing closure and distrust.

This AS, for example, did not comment on the composition of his household; he simply said that even if the conditions in his country were better, he would not want to go back, showing that he had completely cut ties with his family and origins.

I like this life… I don’t want to come back in my country (Nigeria, 31)

I was forced to run away because my uncle put me in this secret society because otherwise, I would not be considered a man. I do not want to see him anymore! (Sierra Leone, 18)

On the other hand, it is true that some of the interviewed ASs brought a renewed positive spin to the future, demonstrating their strong resilience.

Bring them to Italy is my dream. (Gambia, 26)

Profiles like these show that ASs want to maintain a link with their families and to recompose, at least in part, their family nucleus also in Italy once they have obtained the economic stability through work and a contract of employment.

The rupture of family and community bonds represents a risk factor for some ASs; those who instead preserve a positive memory of their place of origin see the link with their past as a source of hope and resources.

He says he would go back to Cameroon if things went well, because anyway it’s his country… He says he still feels good (in Italy) because they treated him well and he cannot complain about this project…he would like to bring also his family. (Cameroon, 29)

### The Host Country and the Relationship with Professionals

The second superordinate theme explored describes the landing in Italy and the relation with the refugee center and the professionals. The new context seems to be represented as a safe place that is able to guarantee a more stable phase.

I remember very well when I arrived, the look I had and the look I have now. If I could find a job I would want to stay here quietly without problems because until now I had everything I need, no one disturbs me, everyone is quiet, and I would like to stay here. (Nigeria, 29)

This AS, showing a great capacity for introspection, gives us a deep reflection on the difference between his self-image at the moment of landing in Italy and the image he had of himself months after landing. This awareness seems to suggest that a process of identity reconstruction is taking place. The serenity (*“everyone is quiet here”*) that comes from the new context that is perceived as welcoming and protective seems to support this process. So, if on the one hand the new context can be a challenge with respect to integration, on the other it is also perceived as a more stable situation in the face of the terrible experiences lived in the land of origin. In other words, a positive perception of the new context represents an important protective factor.

The moment of the Territorial Committee’s evaluation^2^ is experienced as an important turning point in the life of the ASs because it represents a watershed between the old and the new life. All the attention and all the energies of the AS are focused on the outcome of the Committee, but even this moment is not worry-free:

He says that he would prefer to talk about his life in front of a commission because often when he talks about these events, he never feels good; it makes him sad.

So, he hopes, when he goes to the Committee, to be able to tell for the last time everything he has suffered. (Nigeria, 29)

As has emerged from the words of this AS, if the interview with the Committee is experienced as both a source of concern and is also is seen as a “catharsis” during which, for the last time, the individual is “forced” to remember and thus live again the moments of suffering, it opens up the possibility of starting a new life. At this stage of the migratory experience, it is essential to get an identification document.

Work is important, but it’s not that important. These documents are more important. (Senegal, 23)

For this participant, getting the document seems to prevail over everything. It is a priority even over a job search (the primary mission of migrants); the concern for the “document" apparently is associated with recognition and the legitimacy to reside in the country of immigration. Living in a foreign country without documents means not having access to most of the services which all citizens normally access such as the health system and school, and it precludes the possibility of having an employment contract or a lease. For this reason, the quest for the document can lead a situation of high stress for ASs.

The perception of the relationship with professionals, depending on how it is experienced by ASs, can be a source of support or, alternatively, an additional risk factor. In general, regarding the relational dynamics between ASs and operators, the participants put themselves in three different positions: idealization, confidence and conflict.

He also felt the realities of the other refugee centers, and he realizes that anyway here he feels good because the operators also make him understand the importance of the Italian language, and he realizes that everything that operators do is for their own good. (Nigeria, 31)

They treat me very well, but 20 euros per week are few. They are good and help you in many things. When you have a disease, they bring you to the hospital. Even with the documents they helped us, but there were problems. But this is not their fault, it is God. (Senegal, 20)

These ASs demonstrate that they have a realistic view of how operators can help them. If, on the one hand, they recognize the operator’s commitment, they also realize, on the other hand, that operators have a limited margin of action (for example regarding the issue of the document). In other words, they appreciate the value of the work of professionals but recognize that there are limits to actions that depend on reality and do not stem from a lack of willingness.

They did a lot of things for us (…) They’re good. (Nigeria, 31)

The refugee center is good. When I am sick, they take me to the hospital. They give me money, food, and a house. (Senegal, 20)

… operators are like our fathers.’(Cameroon, 29)

These ASs describe the experience within the center in extremely positive terms. The evidence seems to suggest that the guests have idealized the center and the operators who work there.

From our interviews, there were, of course, also episodes of conflict between ASs and professionals. One of the nine ASs in fact told us:

“D. has difficulty with an operator who seems not to take into account the fact that he is an adult and knows very well what to do. According to D, the operator does not consider his needs. He says he tried to talk to him and made him understand that he is 29 years old, almost 30, and he understands very well what he has to do and that he is not forced to do everything the operator wants.” (Cameroon, 29)

From this narration, confidence in the professional’s actions seems to be missing. The AS is struggling to understand the professional’s actions and perceives his authority as a threat or an attack on the relationship. In summary, the relationship with professionals–depending on how it is experienced–can be a source of support or, alternatively, an additional risk factor.

### Future

As previously highlighted, reaching Italian territory was often not a deliberate choice on the part of the participants in our sample. This, in some cases, did not allow for an opportunity to reflect on their expectations about Italy in the period that preceded the landing and during the time spent in the refugee center. Some of the guests, in fact, have shown unclear or negative expectations about Italy:

When I left Nigeria, I had no expectations because first of all I did not think that I would come to Italy. (Nigeria, 29)

It’s too hard to be in Italy. There’s a lot of people here trying to get a job contract, but they don’t pay you, there’s no contract, no document, nothing, it’s hard' (Gambia, 26)

Within the sample, however, we also found those who had clear expectations about the future. Some of these were looking for a job or to study:

I’d like to make pizza, in Italy. (Senegal, 20)

In the future I would like to study engineering… It is the job I want for my future. (Sierra Leone, 18)

Then someone revealed to us:

I would like to marry in Italy and have a family here. With an Italian woman. (Senegal, 23)

The words of this participant express the desire to join the new symbolic order through the desire to build a family with an autochthonous woman. In summary, on one hand ASs cannot imagine their future because is perceived as being full of anguish, yet on the other some have a realistic vision of the future and consider it as being rich with hope that gives the possibility of establishing new bonds.

## Discussion

The representation of origins was the first main theme on which we focused; it appeared to be both a source of trauma and resources for the participants, depending on the meaning they attributed to the experience. The traumatic experiences lived in the lands of origin represent the main motivation that pushed ASs to leave. Additionally, the journey also represents a traumatic event. In this sense, as emerged from the results, the tendency to tell stories which are not affectively mentalized, but which are descriptive and anecdotal, is often common in this kind of population [36–39]. Persecution, discrimination and violence suffered during the journey could represent traumas that are not easy for the migrants to overcome and, even in some cases, not easy to tell. Following Glissant’s [40] interpretation, this attitude could be an expression of what he defines as “the right to opacity”, that is, the right not to be understood and not to totally understand the other. In such a case, the choice not to tell cannot be interpreted as pure discourtesy, reticence or lack of respect towards the researcher; rather, as Glissant points out, it could be attributable to the exercise of a legitimate form of power, perhaps one of the few really available to the ASs. Although participants were aware that the memory of traumatic experiences brought them stress and depression, they were also aware that positive memories about their previous lives could be helpful, connecting them to more positive times, and reminding them of the possibility of a more positive future [19]. In particular, this aspect emerged from the stories about relationships with family. Leaving means taking with you the responsibility for people who remain and whom you have abandoned: parents, relatives, friends, sometimes children and community. When the individual accepts and attributes a value to the original emotional baggage received by his family and is able to elaborate upon even the most critical and painful parts of this relationship, it can count as an important resource, useful to reorganize and consolidate the sense of identity through cultural confrontation with the host society [6].

Even the landing in Italy–in the safe port of the host country—is not an easy time [41]. In fact, at this moment, migrants are asked to retrieve and activate their own resources that can arise basically from the reconstruction of a good image of himself. As emerged from the interviews, this process is favored in good part by a perceived positive atmosphere and serenity in the host context.

The interview with the Committee for the recognition of the right to asylum is a necessary step through which every AS must pass; it can be a cause of stress because the migrant is forced to remember “*for the last time*” the hard experiences lived. This moment is lived also as an act of liberation, a catharsis, becoming the starting point of the new project of the life that they are trying to build in the new country. It also means they have to worry about something more material: to get the document. It is precisely to this purpose that migrants dedicate all their energies.

Another aspect that has been analyzed is the relationship between the professionals and ASs; it can be considered as a resource only when it is based on feelings of confidence and on a realistic perception of what professionals can do in terms of help [42]. The relationships with the operators and the refugee center could lead in some cases to a “dependency”, at the basis of which there may be a form of idealization. This attitude is coherent with one of the specific peculiarities of this population: the tendency towards a constant search for care, of which idealization could be one of the possible outcomes from a compensatory perspective. The operator is seen as a good person, a father, who offers his help without ever asking; to him is delegated the responsibility for the success of the migratory project. This kind of testimony raises the issue of the infantilization of ASs and refugees and the dangerous drift towards a harmful welfarism. The outcome of the relationship with the operators can, unfortunately, also result in overt episodes of conflict as emerged from the story of an AS interviewed. The relationship, in this case, can tun into an additional risk factor.

The last aspect we focused on is the expectation regarding the future. The ASs of our sample reported mainly two types of expectation: absent/negative and positive/realistic. On one hand, the future is negative, unclear and not yet conceivable; expectation had no form because of the lack of preparation regarding the departure and because the refugee experience has not yet been processed. On the other hand, others have shown a renewed proactive drive towards what the new context has to offer in terms of opportunities for study, work or to establish bonds. The desire of one of the participants interviewed to marry an Italian woman seems to show the need to join the new community. This, however, makes us reflect on how much they are prepared to leave behind in terms of renouncing their project of community affiliation, which is the expression of the country of immigration. At the root of this, could be a mimetic tension towards the new cultural order which clearly represents the preferred path to the inclusion process [43]. However, the compulsive willingness to quickly join the new context may be an expression or latent cause of a specific conflict of affiliation by the AS.

We believe that opening a window on the personal history, on the relationship of ASs with professionals and on the representation of the future may help to better understand some deep aspects of the refugee experience. As the results have shown, although the subjects in our sample were exposed to similar experiences in terms of exposure to war-related violence, to instantaneous escapes that do not allow for the planning of a precise migratory project and to the rupture family ties, there are however some differences that allow us to discriminate between the subjects of our sample. In particular, taking into account the different quality of the reported stories and considering that the relational world of ASs could be at the same time a source of risk or a resource, different conditions emerge. The intertwining of the representation of the origins, the relationships with the professionals and the expectations of the future give life to three types of refugee experience that prepare the migrant more or less positively to face the challenge that the new context presents (Table 2).

**Table 2:** The refugee experience

	Origins	Relationship with Professionals	Future	Number of subjects for condition
Persecutory	Persecutory	Negative relationship	Impossibility/difficulty in representing the future	3
Ambivalent	Idealization/devaluation	Relationship of dependency	A future strongly dependent from the reception services	3
Integrated	Positive bondings	Positive/constructive relationship	Positive and realistic expectation towards the future	3

### Persecutory

In this condition, the asylum experience is lived as rather anguished and fragmented. The bond with their origins is not perceived as a protective factor but as strongly persecutory because the migrant has not been able to elaborate upon the deficiency and painful experiences lived in his native land. This condition is characterized by feelings of anger, vindication and an absence of gratitude towards the family and contexts. These feelings may originate from the abuses and the violence caused by the actors met during the journey (i.e., human traffickers in Libya, the leaders of sects or the various protagonists of dictatorial regimes) which are still perceived as living and as a source of danger. Even the relationship with others is particularly compromised in this profile; in fact, the operators of the reception centers can be misunderstood because they can be perceived as threatening or controlling, and this can lead, in some case, to episodes of conflict. The quality of the bonding represents in this case more a constraint than a resource. In light of this distressing interior scenario, the future will also be unthinkable. This naturally has repercussions on the planning of the ASs who might find more difficult to activate—considering the opportunities offered by the new context—their own resources.

### Ambivalent

The feeling that prevails in this condition is ambivalence. This implies a devaluation of everything that happened before the journey and in the native land beside an excessive idealization of what the host context can actually offer or vice versa. We are faced with a condition of strong ambivalence towards the origins and the relationship with the other. The relationship with the other in this case will be less problematic and less conflictual then in the persecutory profile, but there is a high expectation of the Other. Professionals, in fact, are seen as saving figures, “good fathers” who can provide instantly everything the ASs need; unfortunately, this approach can lead to the total absence of assumption of responsibility on the part of the ASs regarding their own migratory project. Although idealization is a powerful way of connection, the more an individual is idealized, the more radical is the devaluation he will face as soon as the other will not be able to live up to the unrealistic expectations to which he was subjected. The expectations regarding the future in this profile are too high or are completely negative. In other words, we are facing a relationship that protects and defends; even if it can repair the pain of what happened at the same time does not provide the resources to encourage the autonomy of ASs and therefore risks creating situations of dependence or rupture. In summary, the delegation to the operators of the migratory project does not allow an adequate activation of the resources that are also present in this condition.

### Integrated

If the subject undergoes a relatively tiring personal experience but at the same time elements of resource, such as feelings of confidence, hope and acceptance are present, we are faced with the integrated profile. The subjects that belong to this condition have a good relationship with their homeland. They recognize the efforts and the pain they have experienced, but at the same time they give value to the original “emotional baggage” they have received from their family. They have a good introspective ability and are able to activate resources that allow them to look at the new context as full of opportunities to seize. Relationships of trust and the feelings of righteousness are their distinctive traits. Moreover, the relationship with operators is seen as a solid starting point on which ASs can actively build their future autonomously. They have a realistic vision of the future that is perceived as a great opportunity. In this condition the resource factors prevail on the risk ones, ensuring the success of the migratory project.

In our sample, three ASs belonged to the persecutory condition, three belonged to the ambivalent condition and three to the integrated one.

### Practical Implications

The three conditions identified can help professionals to better understand, and consequently to orient, between the different experiences of ASs, allowing them to recognize the risks and the resource factors in taking charge of the ASs. In the practice, it should be possible that professionals deal with more Persecutory and Ambivalent profiles, rather than integrated one. As for these two challenging profiles, focusing more on the positive aspects (in terms of opportunities) rather than just on the traumatic ones, can help professionals involved in reception and mental health to not underestimate the less visible aspects of resources—although present in very painful stories—and to develop more effective interventions. This approach shifts the focus from psychopathology to an understanding that relationship aspects have an impact on ASs’ life circumstances and that they may represent an important source of protection. The use of this kind of approach can also assist with the facilitation of an environment that not only recognizes people’s individual resources (resilience) but also their capacity to build bonds as opportunities. Indeed, a discussion about the complexity of migrants' experiences that takes into account both the individual and the relational aspects might prove an excellent platform from which to start interventions to improve professional practices. Care intervention should encourage resources for ASs, such as connections with community organizations, relationships with significant others and a good reintegration related to the migratory experience.

### Limitations and Future Directions

This study suffers some limitations. Firstly, we considered mainly the psychological aspects/internal representations of the ASs refuge experience not collecting information on socio-cultural variables such as education, professional background, level of learning of Italian language, vocational training and work experience. In addition, participants belonged to different ethnicity and, therefore, the study cannot offer a culturally sensitive investigation. Future studies could give more space to these relevant variables.

Secondly, interviews were conducted under the constant supervision of a cultural mediator, and, as such, his presence could in some measure have exerted an influence on the predisposition of the participants to share their personal experiences.

Considering the linguistic and cultural difficulties and the need of the support a consultant, in future studies, it would be interesting to combine the use of different tools that can capture elements at different levels. In this sense, an interesting integration could be provided by the use of graphic symbolic tools. This kind of tool allows for the bypassing of the cognitive area and access to a more unconscious dimension that plays an important role in the exploration of the refugee experience. As the study included only male participants, the results could be biased because it explores migration from a limited perspective. Women have very different experiences of migration characterized by a different threat profile; for this reason, it would be interesting to involve the female population and test whether the three types of refugee experience traced herein are the same for women too.

## Conclusion

This article investigated how a group of ASs hosted in an Italian reception center represents their refugee experience. Using a psychosocial approach that gives importance to the intertwining of personal history and context, the relationships between ASs with their origins, with the host context, with the operators and the representation of the future were explored, looking at aspects related to risks and resources. Our findings highlighted that the bonds with their origins, in particular with their homelands, with their families and the experience of the journey can represent both risk factors and resources depending on the meaning that the ASs attribute to the emotional baggage acquired. At the same time, the relationship with the host context may represent a moment of restart and opportunity or, vice versa, of existential deadlock in different challenges (i.e., the interview with the Commission and the search for the document). The relationship with professionals, in particular, plays a crucial role in the post-migratory experience because it can become a solid base of support on which ASs can rebuild their lives if the relationship based on feelings of trust and is not idealized or particularly conflictual. Finally, there is the possibility of thinking about the future in a positive way, looking at what the new context has to offer in terms of opportunities, or of thinking about a future perceived as distressing and characterized by the absence of bonding. The interweaving of these elements made possible the identification of three different experiences of asylum.

The first type is the persecutory, which is characterized by feelings of anguish, anger and ingratitude towards the family and the place of origin. It is characterized by the absence of bonds or conflicts (such as that with the operators) and by the unthinkability of a future that is lived in a state of anguish. In the ambivalent condition, instead, there are more resource than in the previous one, but they do not guarantee complete stability. In fact, this condition is characterized by the idealization of the bonds with their origins, the host context, the operators and the future. This could be an element of risk because idealization, or its opposite (devaluation), does not ensure the development of personal autonomy, and this can lead to situations of dependence on the reception center and operators. Lastly, the integrated condition represents the optimal one. Despite the efforts that characterize the refugee experience, in this condition ASs are able to activate their resources, which are mostly relational; these are linked to a good representation of their origins, a good relationship with the family, the host country and operators. This condition is characterized by feelings of trust and acceptance that allow individuals to think about the future as rich with opportunities to seize.

In summary, the stories of the migrants are full of affective and relational worlds that accompany and support them in their travels and identity. In the study and in the comprehension of this experience, we cannot ignore the most exquisite relational aspects that characterize it in depth. In conclusion, the refugee experience is a complex and tiring challenge and therefore leads to different outcomes. It can lead to an exchange that allows everyone to recognize themselves and to activate their own personal and relational resources, or vice versa, to a failure that can undermine both physical and mental health.

## Footnotes

1 Before the entry into force of the Security Decree, humanitarian protection was granted to people who did not qualify for political refugee status but who had serious health problems or who came from countries afflicted by natural disasters, for which it was impossible to proceed with a repatriation. In these cases, a return to the country of origin or to a third country would have resulted in the loss of opportunities for care and take-over, which, on the other hand, are guaranteed in Italy.

2 The Territorial Committee is the Italian institution in charge of assessing asylum applications.
